# Carbon and nitrogen availability affects biofilm growth and morphology of the extremotolerant fungus *Knufia petricola*

**DOI:** 10.1128/aem.00656-26

**Published:** 2026-08-28

**Authors:** Abolfazl Dehkohneh, Julia Schumacher, Bastiaan J. R. Cockx, Karin Keil, Tessa Camenzind, Jan-Ulrich Kreft, Anna A. Gorbushina, Ruben Gerrits

**Affiliations:** 1 Department of Materials and the Environment, Bundesanstalt für Materialforschung und -prüfung (BAM), Berlin, Germany; 2 Department of Biology, Chemistry and Pharmacy, Freie Universität Berlin 9166, Berlin, Germany; 3 Department of Analytical Chemistry, Reference Materials, Bundesanstalt für Materialforschung und -prüfung (BAM), Berlin, Germany; 4 School of Biosciences & Institute of Microbiology and Infection, University of Birmingham 1724, Birmingham, United Kingdom; 5 Department of Geosciences, Freie Universität Berlin 9166, Berlin, Germany; Royal Botanic Gardens Kew, Surrey, United Kingdom

**Keywords:** rock-inhabiting fungi, black yeast, subaerial biofilms, growth physiology, Ascomycota, extremophile, melanin

## Abstract

Rock-inhabiting fungi thrive in subaerial oligotrophic environments, such as desert rocks, solar panels, and marble monuments, where organic carbon and nitrogen are scarce. We tested whether the rock-inhabiting fungus *Knufia petricola* showed a preference regarding nitrogen ($NO_{3}^{-}$ or $NH_{4}^{+}$) and carbon (glucose or sucrose) sources, and whether it was sensitive toward carbon and nitrogen limitation. As this fungus produces the carbon-rich, nitrogen-free 1,8-dihydroxynaphthalene (DHN) melanin, we tested whether a melanin-deficient mutant would be less sensitive to carbon limitation. The carbon and nitrogen concentrations were the primary predictors of growth, with a broad optimum partially explained by an optimal fungal C:N ratio. Limiting carbon or nitrogen supply decreased biomass formation, $CO_{2}$ production, and biofilm thickness, but promoted substratum penetration through filamentous growth. The nitrogen content of the biomass was flexible within limits, increasing with increasing nitrogen supply or decreasing carbon supply. The carbon use efficiency was fairly constant, whereas melanization correlated with a higher nitrogen content of the biomass, despite melanin being nitrogen-free. In conclusion, *in vitro*, *K. petricola* switches to explorative growth under nutrient limitations, like fast-growing fungi, revealing universal fungal resource-acquisition patterns.

**IMPORTANCE:**

Since their discovery in the 1980s, extremotolerant fungi have been shown to inhabit desert rocks and coastal salterns, deteriorate marble monuments, weather minerals, colonize solar panels, and positively interact with cyanobacteria, algae, and plant microbiomes. These fungi endure sudden shifts in temperature and water availability, as well as intense sunlight, while persisting on very low nutrient levels. Despite their ecological relevance, their biology remains underexplored. The black fungus *Knufia petricola* offers a rare opportunity to study these organisms in depth because it can be genetically engineered and represents the wider group of resilient surface colonizers. Here, we compared a wild-type strain with a melanin-deficient mutant and generated quantitative data suitable for mathematical modeling of growth. This work provides a foundation for predicting how *K. petricola* behaves on natural and human-made materials, informing future efforts to harness extremotolerant fungi in sustainable technologies, agriculture, and environmental resilience.

## INTRODUCTION

Air-exposed oligotrophic habitats (i.e., subaerial habitats) are characterized by chronically low concentrations of bioavailable nutrients, particularly carbon and nitrogen (1, 2). In such settings, organic carbon can originate from dust—typically richer in organic carbon than the parent soil from which the dust originated (3)—as well as aerosols (4), rain (5), volatile organic compounds (6), pollen (7), or phototrophic symbionts. Nitrogen can be delivered through rain and aerosols (8), gaseous ammonia in agricultural settings (9), gaseous nitrate in urban settings (10), or nitrogen-fixing cyanobacteria (11). However, carbon and nitrogen availability remains low because these surfaces have minimal *in situ* primary production, episodic and small external inputs, and low sorption/retention of organics and ammonium on mineral phases, such that microbial demand frequently exceeds supply (1, 2, 12, 13). Fungi, able to deploy morphological and physiological adaptations, are among the main colonizers of these surfaces, enabling them to affect several biogeochemical cycles (1, 2, 14).

One group of fungi able to colonize these oligotrophic, subaerial surfaces are extremotolerant rock-inhabiting fungi (RIF), which can be isolated from surfaces like roof tiles (15, 16) and marble monuments (17). RIF are adapted to survive the multiple stresses typical for these environments, such as nutrient scarcity (1, 18) and osmotic, radiation, and oxidative stresses (19–22). These fungi typically display three morphologically distinct forms of growth: yeast-like, meristematic, and (pseudo)hyphal (23–25), between which they may switch depending on environmental conditions (26). RIF are furthermore characterized by their generally slow growth and constitutive production of dihydroxynaphthalene (DHN) melanin (1, 21, 25). This black pigment, mostly incorporated into the outer cell wall, provides resistance to several stresses (20, 27, 28).

Most fungi can assimilate a wide range of nitrogen and carbon sources. Simple carbohydrates, particularly glucose, are more easily utilized than more complex sugars (29). Glucose supports faster growth due to its rapid uptake and more direct entry into central metabolic pathways (30, 31). Ammonium is typically prioritized over nitrate (32), as the reduction of nitrate to ammonium requires eight electrons (reducing ATP production in the respiratory chain) and the synthesis of additional enzymes (33, 34).

The carbon-to-nitrogen (C:N) ratio of the medium is a primary determinant of fungal growth, with species-specific optima for biomass production (35, 36). Increasing the C:N ratio creates nitrogen scarcity that redirects resources toward nitrogen acquisition. This shift has been shown to increase mycelial extension (35), reduce mycelial density (35), and lower overall biomass yield (37). These *in vitro* findings contrast with field studies, where fungal abundances generally decrease with increasing nitrogen additions to soils (38–40). Such apparently negative responses of soil microbes to nitrogen additions are potentially driven by indirect effects, i.e., shifts in pH or soil-plant interactions (41), making the interpretation of microbial nutrient limitation studies challenging. How carbon and nitrogen sources and their relative proportions affect growth and biofilm morphology and whether melanin influences physiological adaptation to nutrient conditions beyond stress resistance in extremotolerant black fungi remains poorly understood.

Here, we aimed to address these gaps by focusing on *Knufia petricola*, a model for RIF with a sequenced genome and available genetic engineering tools allowing functional genetics studies (42–45). *K. petricola* can assimilate 63 out of 190 tested carbon and 71 out of 95 tested nitrogen compounds, including various sugar alcohols, mono-aromatics, and amino acids (46). Here, we compared growth on sucrose with glucose, as both are secreted by the photobionts of lichens (47). For nitrogen, we selected nitrate and ammonium as atmospheric nitrogen sources typical for urban and agricultural settings, respectively. We evaluated how different concentrations (and thus varying C:N ratios) of these carbon and nitrogen sources affected growth parameters, such as penetration depth, biomass production, and carbon use efficiency (CUE). We further evaluated whether the formation of carbon-rich, nitrogen-poor melanin affected these nutrient-dependent growth characteristics by comparing the melanized wild type (WT) with a non-melanized mutant (∆*pks1*). We hypothesized that *K. petricola,* as an extremotolerant fungus, would be able to grow at low carbon and nitrogen supplies, would have no preferences for the types of carbon or nitrogen sources, and, if melanized, would be more sensitive to carbon limitation. In short, we found that carbon and nitrogen limitation affected the growth characteristics of *K. petricola* most strongly, while melanin formation and the type of carbon or nitrogen source were less important.

## MATERIALS AND METHODS

### General experimental design

The effects of glucose (Glc) and sucrose (Suc) as carbon sources were tested in combination with $NaNO_{3}$ (hereafter referred to as $NO_{3}^{-}$) and $NH_{4}Cl$ (hereafter referred to as $NH_{4}^{+}$) as nitrogen sources. The molar C:N ratios were adjusted by manipulating the carbon or the nitrogen concentration of a defined minimal medium; these conditions are hereafter referred to as the carbon and nitrogen experiments. In the carbon experiment, the $NO_{3}^{-}$ and $NH_{4}^{+}$ concentrations were kept at 10 mM while the Glc or Suc concentrations were varied (Table 1). In the nitrogen experiment, the carbon concentration was kept constant, at 100 mM for Glc or 50 mM for Suc, while the nitrogen supply was varied (Table 1). The medium was supplemented with the following salts and trace elements, at final concentrations of 5.88 mM ${KH}_{2}PO_{4}$, 6.71 mM KCl, 4.15 mM $MgSO_{4}$, 0.90 mM $CaCl_{2}$, 0.16 µM $H_{3}BO_{3}$, 0.025 µM $MnCl_{2}$, 7.55 µM $FeSO_{4}$, 0.80 µM $CoCl_{2}$, 0.10 µM $NiCl_{2}$, 0.059 µM $CuCl_{2}$, 0.50 µM $ZnSO_{4}$, 10 µM NaOH, 0.023 µM $Na_{2}SeO_{3}$, and 0.149 µM $Na_{2}MoO_{4}$, diluted in ultrapure water (Milli-Q). Five control media were prepared in which the carbon source, the nitrogen source, or both the carbon and nitrogen sources were omitted (Table 1). The pH was adjusted to 5. Media were solidified by adding 15 g L^−1^ bacteriological agar (AppliChem) and sterilized via autoclaving.

**TABLE 1:** Concentration of nutrients in the carbon and nitrogen experiments^*a*^

C:N ratio (molar)		0.6	6	60	600	6,000	Controls		
Carbon experiment							C−/ $NO_{3}^{-}$	C−/$NH_{4}^{+}$	C−/N−
C	Glc (mM)	1	10	100	1,000		0	0	0
	Suc (mM)	0.5	5	50	500		0	0	0
N	$NO_{3}^{-}$ (mM)	10	10	10	10		10		0
	$NH_{4}^{+}$ (mM)	10	10	10	10			10	0
Nitrogen experiment							Glc/N−	Suc/N−	
C	Glc (mM)	100	100	100	100	100	100		
	Suc (mM)	50	50	50	50	50		50	
N	$NO_{3}^{-}$ (mM)	1,000	100	10	1	0.1	0	0	
	$NH_{4}^{+}$ (mM)	1,000	100	10	1	0.1	0	0	

^*a*^ In the carbon experiment, the concentration of the nitrogen source, either $NO_{3}^{-}$ or $NH_{4}^{+}$, was constant at 10 mM, and the concentration of carbon was varied from 1 mM to 1 M for Glc and 0.5 mM to 500 mM for Suc. In the nitrogen experiment, the carbon concentration was constant, either 100 mM for Glc or 50 mM for Suc, while nitrogen concentration was varied from 0.1 mM to 1 M. Conditions which were not tested are shown by a gray background.

### Strains and culturing

Experiments were performed with *K. petricola* strain A95, isolated from a marble monument in Athens, Greece (17). The melanin-deficient mutant ∆*pks1* (KP-0033, transformant PN3) was described previously (42). Prior to experiments, the strains were inoculated onto solid minimal medium containing 100 mM Glc and 10 mM $NO_{3}^{-}$ (i.e., a C:N ratio of 60) and incubated for 7 days at 25°C in darkness. The fungal biofilms were collected and, together with 500 µL ultrapure water and 4–5 glass beads (3 mm diameter), mechanically dispersed for 5 min using a mixer mill (Retsch) set at 30 s^−1^. Colony-forming units (CFU) in the suspensions were determined using a Thoma cell counting chamber and adjusted to a final titer of 10⁷ CFU mL^−1^. Ten microliters of these inocula were separately dropped onto 12.5 mL of solid medium in 6 cm Petri dishes and incubated for 28 days at 25°C in darkness.

Although we aimed to cover a wide range of carbon and nitrogen concentrations, the lower experimental carbon and nitrogen concentrations are within the range of field data. Vogel et al. (7) measured the total organic carbon (TOC) and nitrogen (TN) deposition from the atmosphere in three locations over 14-day periods. The differences between the locations were rather large; the concentration of TOC lay in an approximate range of 25 to 7,000 mg m^−2^ (50 to 14,000 mg m^−2^ for 28 days), and the concentration of TN was approximately 0 to 130 mg m^−2^ (0 to 260 mg m^−2^ for 28 days). The carbon amounts in our Petri dishes ranged from 0.9 to 900 mg, whereas the nitrogen amounts ranged from 0.017 to 170 mg. Considering a Petri dish surface area of 28 cm^2^, this corresponds to concentration ranges of 321 to 321,000 mg m^−2^ for carbon and 6.1 to 61,000 mg m^−2^ for nitrogen. The lower concentrations from our experiment are therefore within the range measured by Vogel et al. (7).

### Biofilm morphology

On day 28, micrographs of the biofilms and their cross-sections were taken using a digital camera and a stereomicroscope (Stemi 2000-C, Carl Zeiss). Cross-sections (~2 mm thick) were cut out from the middle of the biofilms using a scalpel and placed on microscope slides for imaging. Cross-section images were used to measure the maximal thickness of the biofilm and the maximal depth of visible penetration into the agar. For this purpose, the agar surface next to the biofilm was used as a reference height relative to which thickness and penetration were quantified. When biofilm growth pushed the agar upward, the upper surface of this agar was used as the reference height.

**Fig 1:**
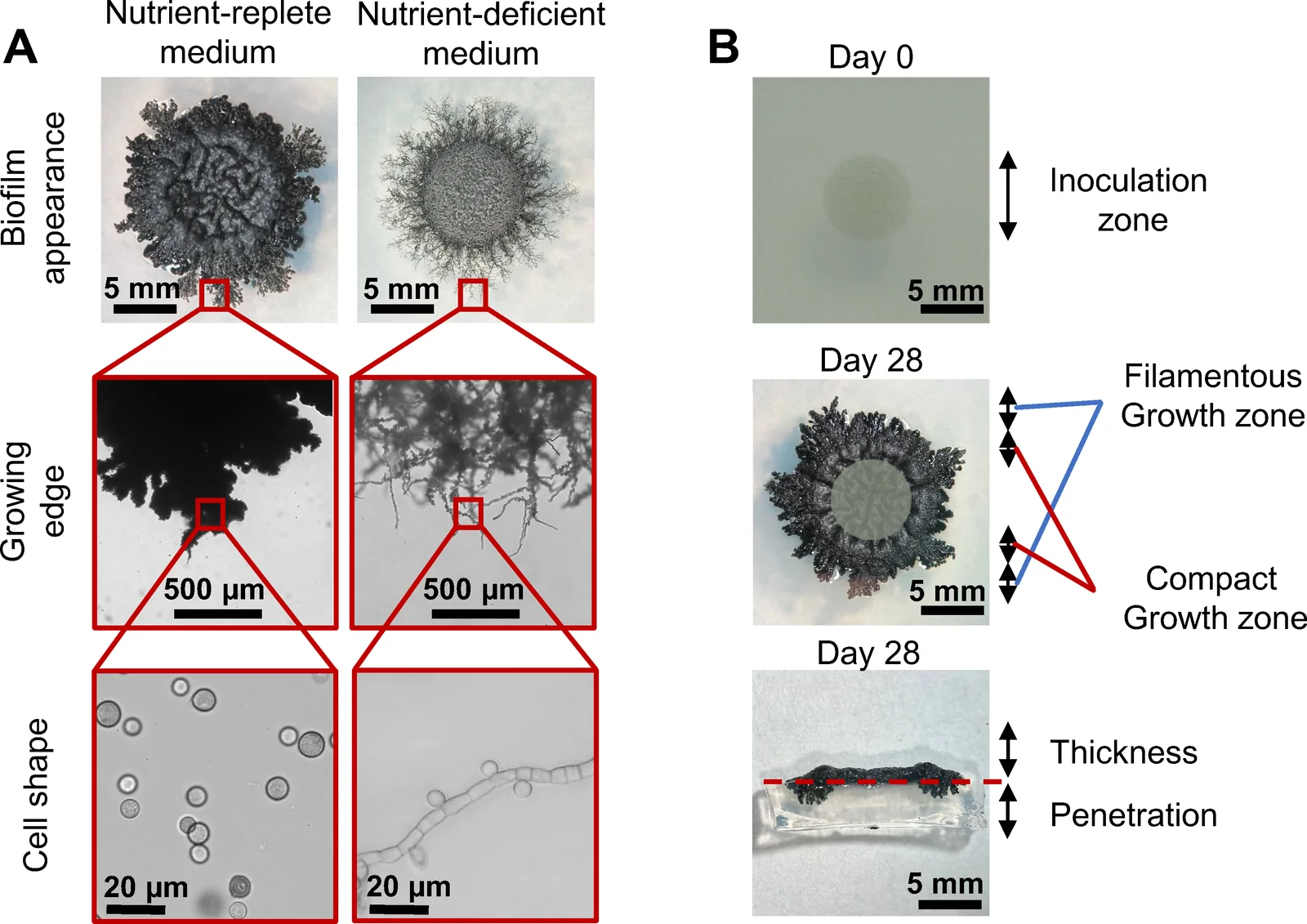
The morphology of *K. petricola* biofilms and cells depended on nutrient availability. Droplets containing 10^5^ CFU of *K. petricola* (WT) were spotted onto solid media with defined nutrient contents and incubated for 28 days at 25°C in the dark. (**A**) Biofilms formed in nutrient-replete (100 mM Glc and 10 mM $NO_{3}^{-}$) or nutrient-deficient (1 mM Glc and 0.1 mM $NO_{3}^{-}$) conditions. Starvation caused more filamentation around the biofilm edge. Yeast-like cells were observed in nutrient-replete conditions, while more filaments were present in nutrient-deficient media. (**B**) Definition of growth zones. The maximum sizes of the inoculation zone and the compact and filamentous growth zones were measured to study the effects of nutrient availability on biofilm morphology. Penetration depth and biofilm thickness were quantified relative to the agar surface adjacent to the biofilm. Growth above this reference plane was recorded as biofilm thickness, whereas growth below was recorded as penetration depth.

The biofilm size was quantified using the Feret diameter, the longest distance between any two points on the biofilm edge, via ImageJ (48). This was measured for three distinct zones of the biofilms: the innermost inoculation zone, the surrounding zone with compact growth, and the outermost zone with filamentous growth (Fig. 1). The inoculation zone is the extent of the biofilm measured after inoculation (day 0). The compact growth zone is dense and lacks visible filaments, and the filamentous growth zone is situated around the compact growth zone and characterized by the presence of filaments (Fig. 1).

### Biofilm biomass

For determining the biomass on day 28, biofilms were scraped from the agar using a scalpel and transferred separately to bottles containing 50 mL of ultrapure water. The bottles were microwaved for 5–7 min to melt the agar. The resulting mixture was poured into 50 mL tubes, and the biofilm was allowed to separate from the aqueous agar solution. Once the biofilm settled at the bottom (no centrifugation necessary), the supernatant (i.e., water and dissolved agar medium) was discarded, and the biofilm was washed twice with hot water to remove residual agar. The biofilms were transferred to 2 mL polypropylene microcentrifuge tubes and dried in a heating cabinet at 65°C for 2 days. Subsequently, the tubes were equilibrated to room temperature, and the dry biomass was weighed. Partial loss of filaments could have occurred during the unavoidable heating and washing steps, but most of the biomass was concentrated in the denser zones and therefore unlikely to get lost.

### Biofilm carbon and nitrogen contents

After weighing, the biomass samples were used for elemental analysis, pooling two technical replicates within each condition prior to measurement. Carbon and nitrogen contents were determined using an elemental analyzer (EA 3100, Eurovector) based on the Dumas combustion technique. Approximately 1–2 mg of biofilm sample was encapsulated in a tin capsule and subjected to dynamic flash combustion at 950°C in a stream of pure oxygen. The resulting combustion gases ($N_{2}$, $CO_{2}$, and $H_{2}O$) were passed through a reduction column and a moisture trap, followed by gas chromatographic separation and detection using a thermal conductivity detector. Elemental concentrations were quantified using the Weaver software supplied with the elemental analyzer (Eurovector).

### Respiration and carbon use efficiency

Respiration was measured via regular $CO_{2}$ concentration analysis of the headspace of sealed 100-mL glass vials containing 12.5 mL solid medium (as for the Petri dish experiments) and the fungal inoculant. The cultures were incubated for 10 days in darkness at 25°C. The amount of $CO_{2}$ gas produced was measured on days 1, 2, 3, 4, 7, 8, 9, and 10 using a LI-6400XT system (LI-COR). To avoid $O_{2}$ limitation and $CO_{2}$ accumulation, the vials were flushed for 5 min with ambient air that was rendered $CO_{2}$-free by passing it through a soda-lime $CO_{2}$ scrubber prior to entering the vials. Dissolved inorganic carbon was estimated from measured headspace $CO_{2}$ concentrations using Henry’s law (49) at 25°C, treating dissolved $CO_{2}$ and carbonic acid ($H_{2}CO_{3}$) as a single species. The partitioning between dissolved $CO_{2}$ and bicarbonate ($HCO_{3}^{-}$) was calculated using the apparent first dissociation constant of carbonic acid (pK_a1_ = 6.35) assuming that the pH was 4, as *K. petricola* can acidify the medium (50). Repeating the calculation for other pH values (i.e., 3 and 5) hardly changed the results. Total $CO_{2}$ production was calculated as the sum of headspace $CO_{2}$ and dissolved inorganic carbon. The CUE was determined by dividing the carbon assimilated into biomass on day 10 (B) by the total carbon (R + B) that was either respired by or assimilated into the biomass according to the formula described by Maynard et al. (51): CUE=B/(R+B). For some conditions, due to poor growth within 10 days, the CUE could not be calculated.

### Statistical analysis

Most figures were created in Origin 2023. Asterisks in figures and tables indicate significance levels: * for *P* < 0.05, ** for *P* < 0.01, and *** for *P* < 0.001. All quantitative values are presented as mean ± standard error (SE). All statistical analyses and model visualizations were conducted in R (4.3.0) using the following R packages: readxl, ggplot2, plotly, corrplot, polycor, FactoMineR, GenSA, MASS, htmltools, dplyr, tidyverse, and tidyr.

The data set consisted of three biological replicates for each treatment combination in each experiment. The categorical variables strain, nitrogen source, and carbon source were treated as factors, while carbon and nitrogen concentrations or the C:N ratio were treated as quantitative variables. Quadratic terms for carbon and nitrogen concentrations or the C:N ratio were included to capture non-linear relationships expected from visual exploration of the data. To assess the influence of these explanatory variables on biofilm responses, distinct regression models were applied for each response variable. These initial, full regression models included all main effects and all possible interaction terms. Stepwise model reduction was performed in both forward and backward directions based on minimizing the Akaike Information Criterion (AIC) to identify the most parsimonious model. Model residuals were tested for normality, homoscedasticity, and independence to check the validity of assumptions.

First, we compared models using either the C:N ratio or its log_10_-transformed values (including quadratic terms and interactions with strain, nitrogen, and carbon treatment) as predictors. These models were compared (i) using statistical measures and (ii) physiological appropriateness. While the untransformed C:N ratio model achieved marginally higher adjusted *R*^2^ for a few state variables, the shape of the fitted model indicated unphysiological predictions outside the data range and more extreme peaks and curvatures suggesting overfitting (10.5281/zenodo.18980984). Specifically, the model based on untransformed C:N ratios generated anomalous predictions (e.g., high peaks at C:N ratios ~300, followed by steep declines). The log_10_-transformed C:N model produced more realistic, broader, and lower maxima rather than extreme behavior, and therefore log-transformed data were used for further analysis.

Second, we compared two approaches before deciding on the most appropriate method. The first approach was to use the C:N ratio as an “integrative” explanatory variable (called ratio-based models), and the other was to use carbon and nitrogen concentrations as separate explanatory variables (called concentration-based models). The former approach resulted in different regression models for both the carbon and nitrogen experiments, which made interpretation difficult as different optima were obtained for the carbon and nitrogen experiments. Moreover, although some response variables were heteroscedastic, the overall residual distributions were closer to the expected patterns for normal and homoscedastic variables for the concentration-based models. Note that any systematic deviations from expected patterns were toward the ends of the range, which would not affect the regions around the optima. We therefore decided to use concentration-based models based on log_10_-transformed concentrations as the most appropriate models, and these results are presented in the main text; for results from the other models, see 10.5281/zenodo.18980984.

## RESULTS

### Nutrient availability and type of nitrogen source controlled single-cell and biofilm morphology

**Fig 2:**
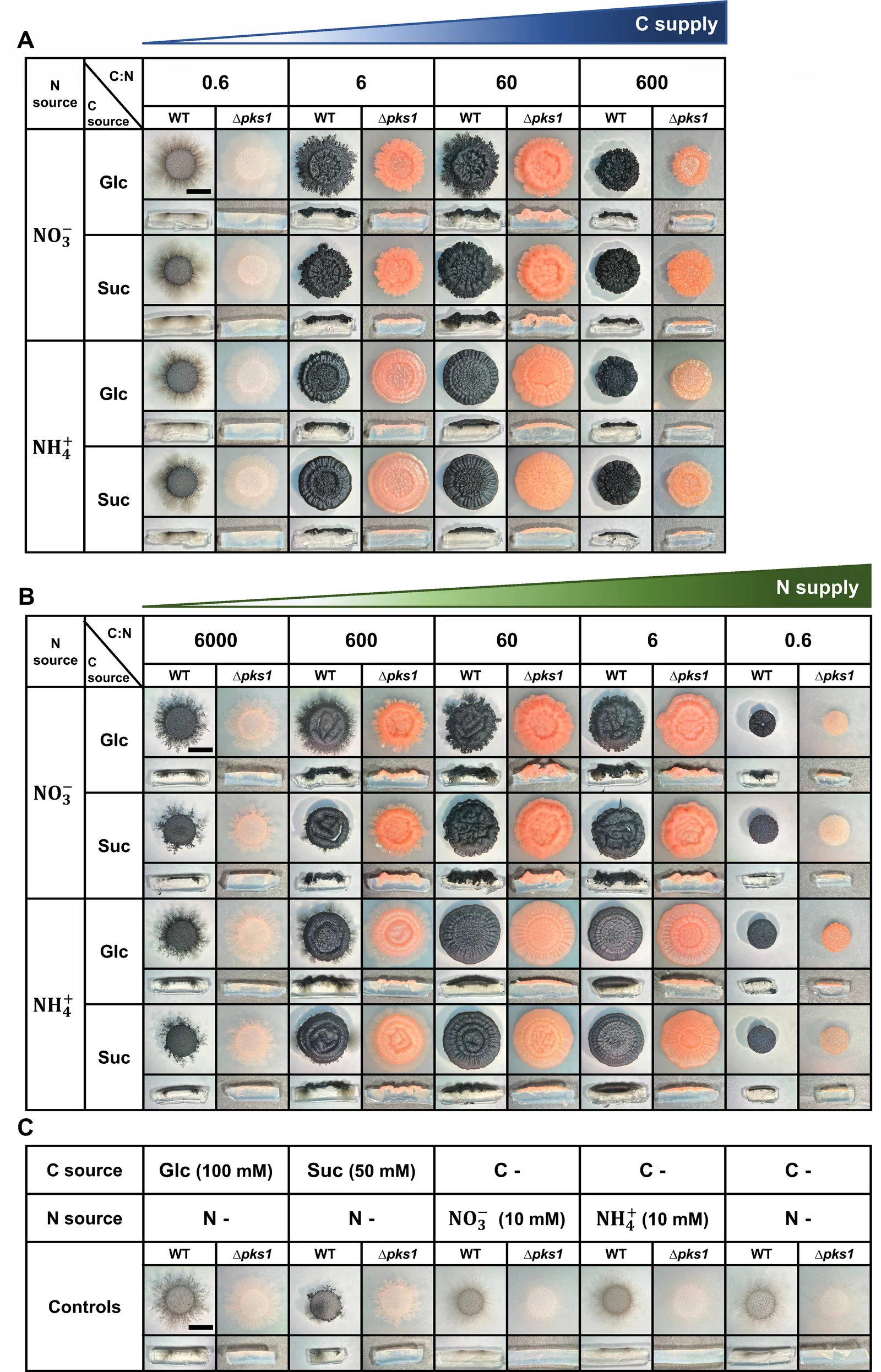
Nutrient levels and nitrogen source determine biofilm morphology of *K. petricola* strains. One droplet containing 10^5^ CFU of the WT or the melanin-deficient ∆*pks1* mutant was dropped on the solid medium and incubated for 28 days at 25°C in the dark. All experiments were conducted in triplicate; images of representative biofilms (top views and cross-sections) are shown. Scale bars represent 5 mm. (**A**) The effect of carbon availability (designated as the carbon experiment) was tested by increasing the glucose (Glc) concentration from 1 mM to 1 M or sucrose (Suc) from 0.5 to 500 mM, keeping $NH_{4}^{+}$ or $NO_{3}^{-}$ concentrations at 10 mM, corresponding to molar C:N ratios of 0.6 to 600. (**B**) The effect of nitrogen availability (designated as the nitrogen experiment) was tested by increasing the concentration of $NO_{3}^{-}$ or $NH_{4}^{+}$ from 0.1 mM to 1 M, keeping the concentrations of Glc at 100 mM or Suc at 50 mM, corresponding to molar C:N ratios of 6,000 to 0.6. (**C**) The effect of starvation was tested in unsupplemented media (C−/N−) or in media supplemented with a carbon or nitrogen source only.

The concentration of carbon and nitrogen affected both biofilm and single-cell morphology of *K. petricola*. At higher concentrations of both nutrients, the biofilm was compact and consisted of yeast-like, spherical cells, while at lower concentrations, filamentous growth consisting of pseudo-hyphal structures was observed surrounding the compact growth zone (Fig. 1). The nitrogen source affected the biofilm shape, regardless of the carbon source (Fig. 2). In the presence of $NO_{3}^{-}$, biofilms were thicker at the edge, had small folds in the center, and more filamentation, while in the presence of $NH_{4}^{+}$, they had a smoother surface and edge and less filamentation (Fig. 2A and B). However, at low carbon or nitrogen concentrations (C:N ratio of 0.6 in the carbon and 6,000 in the nitrogen experiment), the biofilm morphology was similar (Fig. 2). The biofilms of the WT and ∆*pks1* mutant had similar morphologies. Withholding carbon, nitrogen, or both triggered filamentation around the compact growth zone (Fig. 2C). Increasing the supply of nitrogen while keeping Glc at 1 mM did not affect biofilm morphology, whereas increasing the carbon concentration while keeping $NO_{3}^{-}$ at 0.1 mM resulted in denser biofilms (Fig. S1).

**Fig 3:**
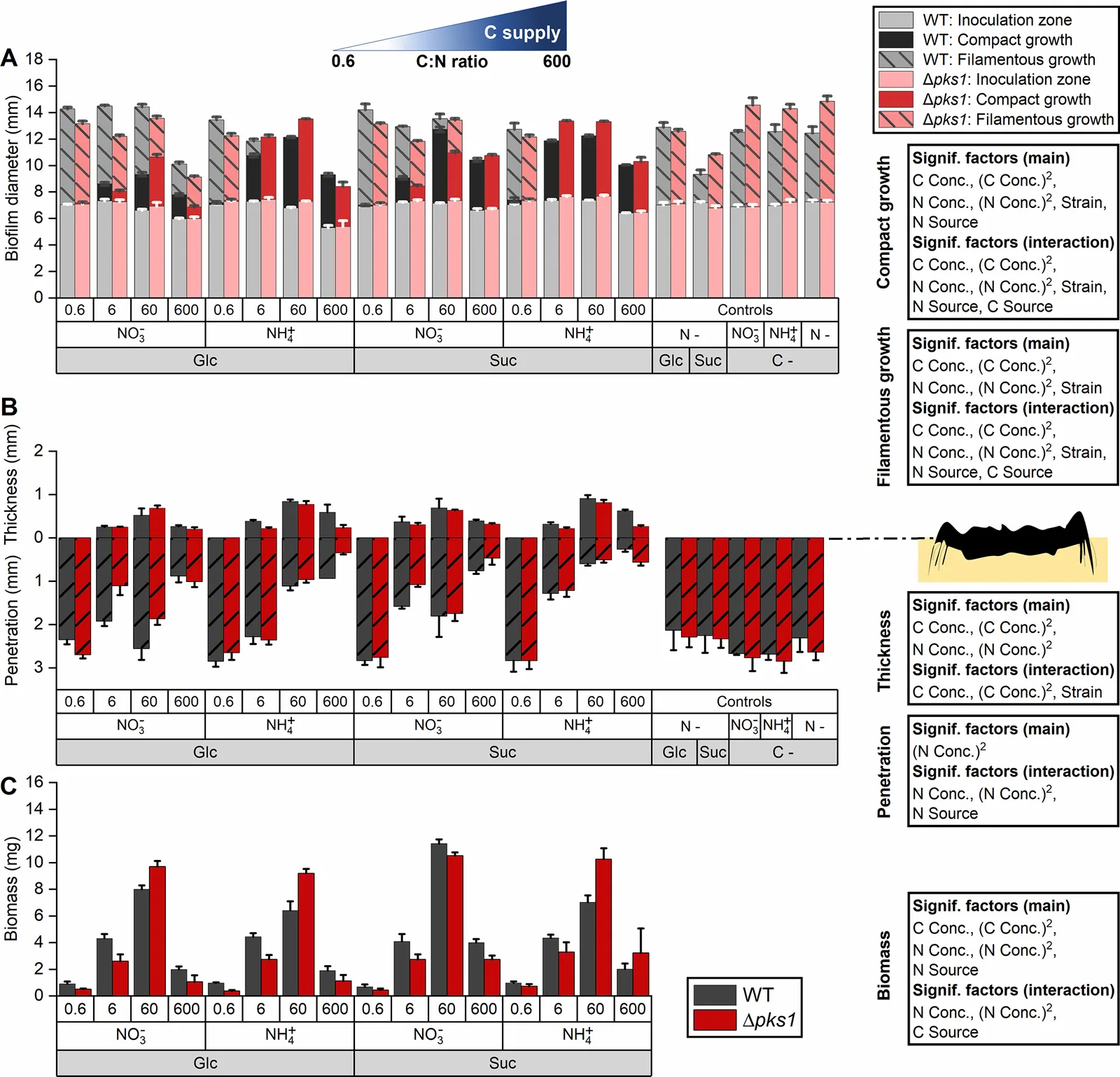
Effects of increasing carbon supply (the carbon experiment) on filamentation, substrate penetration, biofilm thickness, and biomass for WT and *∆pks1* mutant after 28 days. (**A**) Increasing the carbon concentration reduced filamentation and increased compact growth (both based on maximum diameter of respective zones). Absence of either carbon or nitrogen led to biofilms expanding via filamentation. (**B**) Biofilms grown on media with lower concentrations of carbon were thinner and penetrated the agar more deeply. (**C**) Biomass was highest at the intermediate C:N ratio of 60. The amount of biomass in the controls was too low to be measurable. All experiments were conducted in triplicate; error bars show SE. Statistical significance is shown for the main variables and for the interactions among these variables based on the most parsimonious regression models (Table 2).

**Fig 4:**
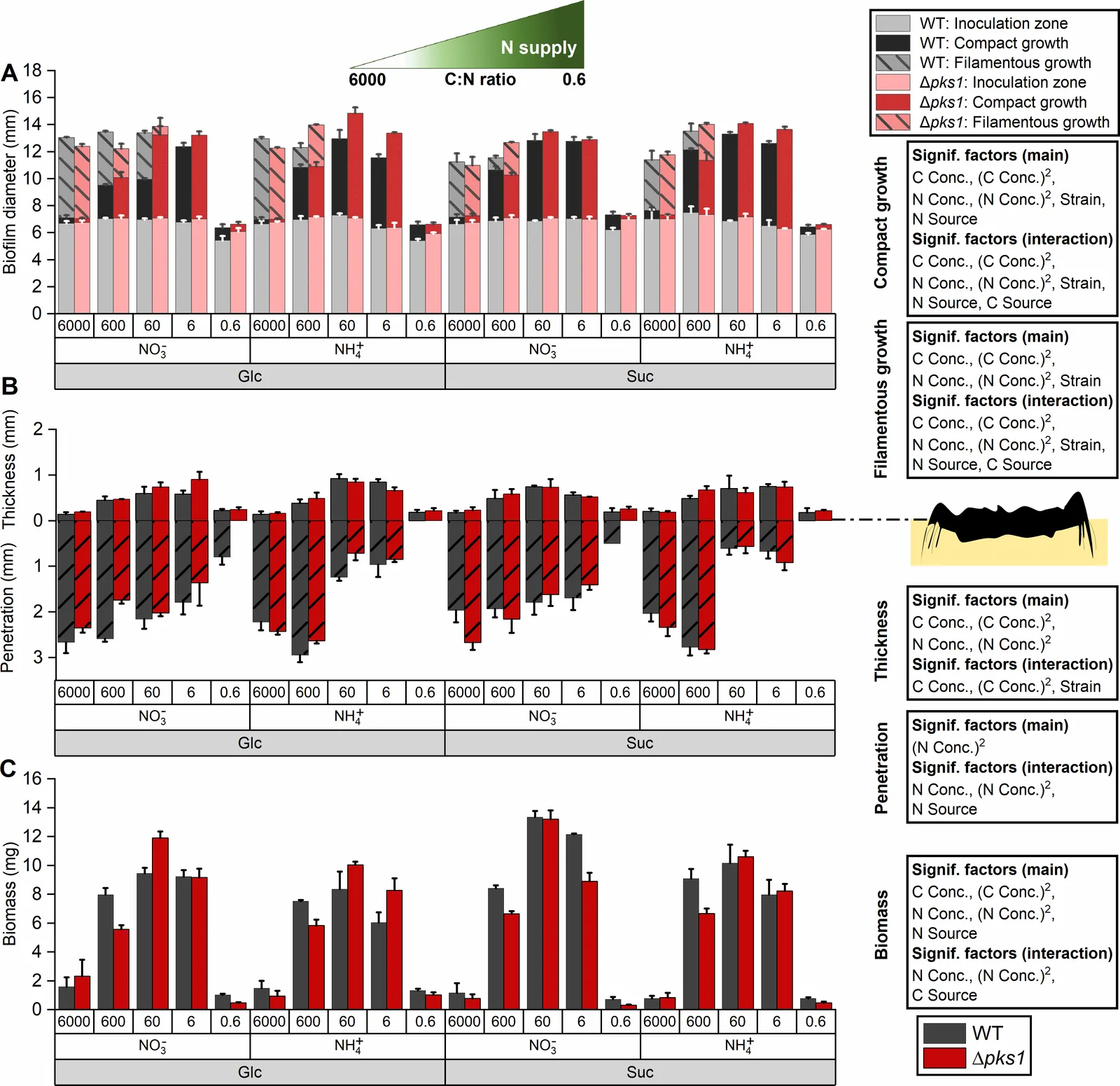
Effects of increasing nitrogen supply (the nitrogen experiment) on filamentation, substrate penetration, biofilm thickness, and biomass for WT and ∆*pks1* mutant after 28 days. (**A**) Increasing nitrogen concentration reduced filamentous growth and increased compact growth (both based on maximum diameter of respective zones). (**B**) Biofilms penetrated the agar more deeply at lower nitrogen concentrations, while thickness increased with increasing nitrogen. (**C**) The highest biomass was formed at the intermediate C:N ratio of 60. All experiments were conducted in triplicate; error bars show SE. Statistical significance is shown for the main variables and for the interactions among these variables based on the most parsimonious regression models (Table 2).

The filamentous and compact growth zones increased and decreased, respectively, upon carbon or nitrogen limitation (Fig. 3A and Fig. 4A). The most parsimonious of the concentration-based regression models with log_10_-transformed carbon and nitrogen concentrations showed that the linear and quadratic terms of both carbon and nitrogen concentrations were strong predictors for both growth zones (Table 2; Table S1). Unless stated otherwise, variables were significant. When carbon, nitrogen, or both were absent, biofilms expanded exclusively through filamentous growth, with no compact growth zone (Fig. 3A and Fig. 4A). The presence of $NO_{3}^{-}$ altered the nitrogen concentration-dependent response and promoted filamentous growth at intermediate nitrogen concentrations (Fig. 3A and Fig. 4A; Table S1), whereas $NH_{4}^{+}$ favored compact growth regardless of the nitrogen concentration (Table 2). Moreover, the WT produced more filaments than the mutant in $NH_{4}^{+}$ and Glc; however, this effect depended on the nutrient concentrations (Fig. 3A and Fig. 4A; Table 2; Table S1).

**TABLE 2:** Summary of the most parsimonious concentration-based regression models for biofilm growth responses to explanatory variables^*a*^

	Intercept	Log (C atoms)	(Log (C atoms))^2^	Log (N atoms)	(Log (N atoms))^2^	Strain	Nitrogen source	Carbon source	AIC	Adjusted *R*^2^
Thickness	***	***	***	***	***				−755	0.689
Penetration	***				***				−261	0.719
Compact growth zone	***	***	***	***	***	*	*		64	0.815
Filamentous growth zone	***	***	***	***	***	*			−75.7	0.893
Biomass 28 dpi	***	***	***	***	***		*		336	0.723
C content 28 dpi	***				*		*		677	0.469
N content 28 dpi	***	***	***		***				325	0.863
H content 28 dpi	***					*			341	0.334
Fungal C:N 28 dpi	*	***	***	*		*			338	0.638
CO_2_ 10 dpi	***	***	***	***	***		**		−125	0.917
CUE 10 dpi	***			**	**				262	0.359
C content 10 dpi	***	***	**	***			**	***	415	0.357
Biomass 10 dpi	***	***	***	***	***				−129	0.781

^*a*^ Regression models were fitted to assess the effects of log_10_-transformed atomic concentrations of carbon and nitrogen (linear and quadratic terms) and the factors strain, nitrogen and carbon source on growth. The log_10_-transformed nutrient concentrations were included as both linear and quadratic terms to allow for the apparent non-linear responses. Model selection was performed using stepwise removal and addition of terms to minimize the Akaike Information Criterion (AIC), which identified the most parsimonious model that balanced explanatory power and model complexity. Shown with a gray background are the variables that were removed from this parsimonious model. The intercept represents the predicted response at zero carbon and nitrogen concentrations for the reference levels of strain, nitrogen and carbon sources, and therefore serves as a baseline. Model fit was also evaluated using the adjusted *R*^2^, which accounts for the number of predictors included. Statistical significance is indicated using standard thresholds (*P < 0.05, **P < 0.01, ***P < 0.001). Data represent measurements at 28 or 10 dpi (days post-inoculation). Overall, carbon and nitrogen concentrations and their quadratic terms were the most significant predictors of most growth characteristics. This table shows only the main effects, the full models with all interactions are included in Table S1. For model fitting and more details see the R notebook (10.5281/zenodo.18980984).

While thickness increased with increasing carbon or nitrogen supply, penetration depth showed the opposite trend; however, this was not significant for carbon (Fig. 3B and Fig. 4B; Table 2). Although the main effect of nitrogen source was not significant, its significant interaction with nitrogen concentration shows that the influence of the nitrogen source on penetration depth was concentration-dependent (Table S1), with $NO_{3}^{-}$ promoting deeper penetration under high nitrogen conditions (Fig. 4B; Table 2; Table S1). Overall, the carbon and nitrogen concentrations and their quadratic terms were the key predictors for biofilm morphology, with the nitrogen source having a weaker effect.

### Nutrient availability determined biomass production

**Fig 5:**
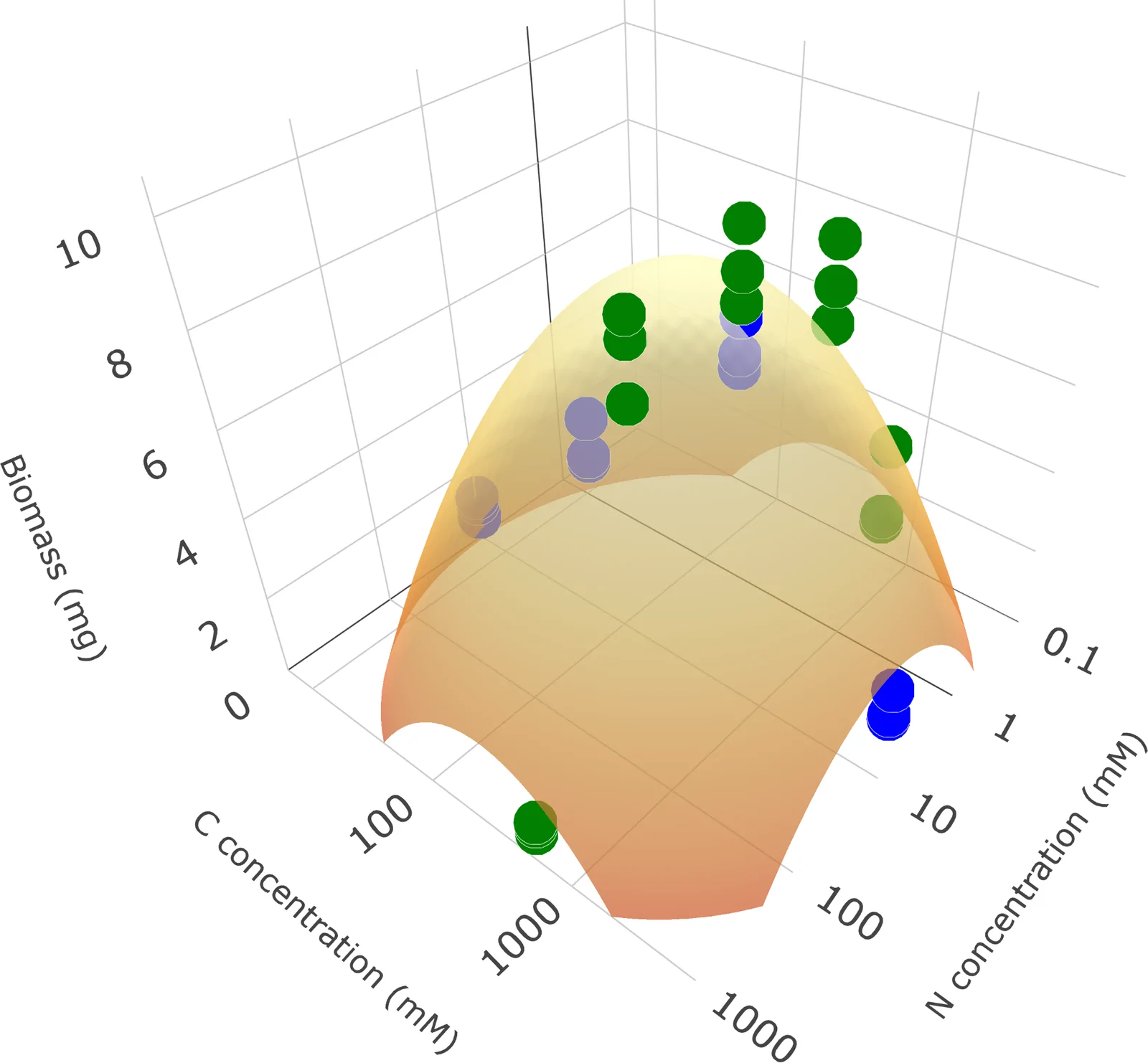
The model-inferred optimal C:N ratio of the medium for biomass growth. The surface visualizes the predictions from the most parsimonious (lowest AIC) concentration-based regression model using log₁₀-transformed atomic carbon and nitrogen concentrations as explanatory variables and biomass after 28 days as response variable. This model incorporated quadratic terms for both carbon and nitrogen concentrations to capture non-linear dose-response relationships. In this model, the carbon and nitrogen concentrations and their quadratic terms and the nitrogen source had a significant effect on the biomass (Table 2; Table S1). The dots show experimentally observed biomass values from both the carbon (blue) and nitrogen (green) experiments for the WT grown on minimal medium with Glc and $NO_{3}^{-}$ as the sole carbon and nitrogen sources. The surface shows the model prediction under the same conditions. Axes display log₁₀-scaled carbon (C) and nitrogen (N) concentrations (mM), the z-axis indicates biomass (mg). The model predicts a broad biomass maximum at a C:N ratio of 26.9 (273 mM carbon and 10.1 mM nitrogen).

Biomass was highest at intermediate nutrient concentrations (Fig. 3C and Fig. 4C; Table 2). Biomass varied less for the nitrogen than for the carbon experiment within the C:N ratio range of 6 to 600. Using the most parsimonious concentration-based regression model with log_10_-transformed carbon and nitrogen concentrations, the optimal biomass was inferred to be at 273 mM carbon and 10.1 mM nitrogen, i.e., a medium C:N ratio of 26.9, which is between the observed C:N ratios of 6 and 60 (Fig. 5; Table S1).

### Biofilm carbon content was constant while nitrogen content was flexible

**Fig 6:**
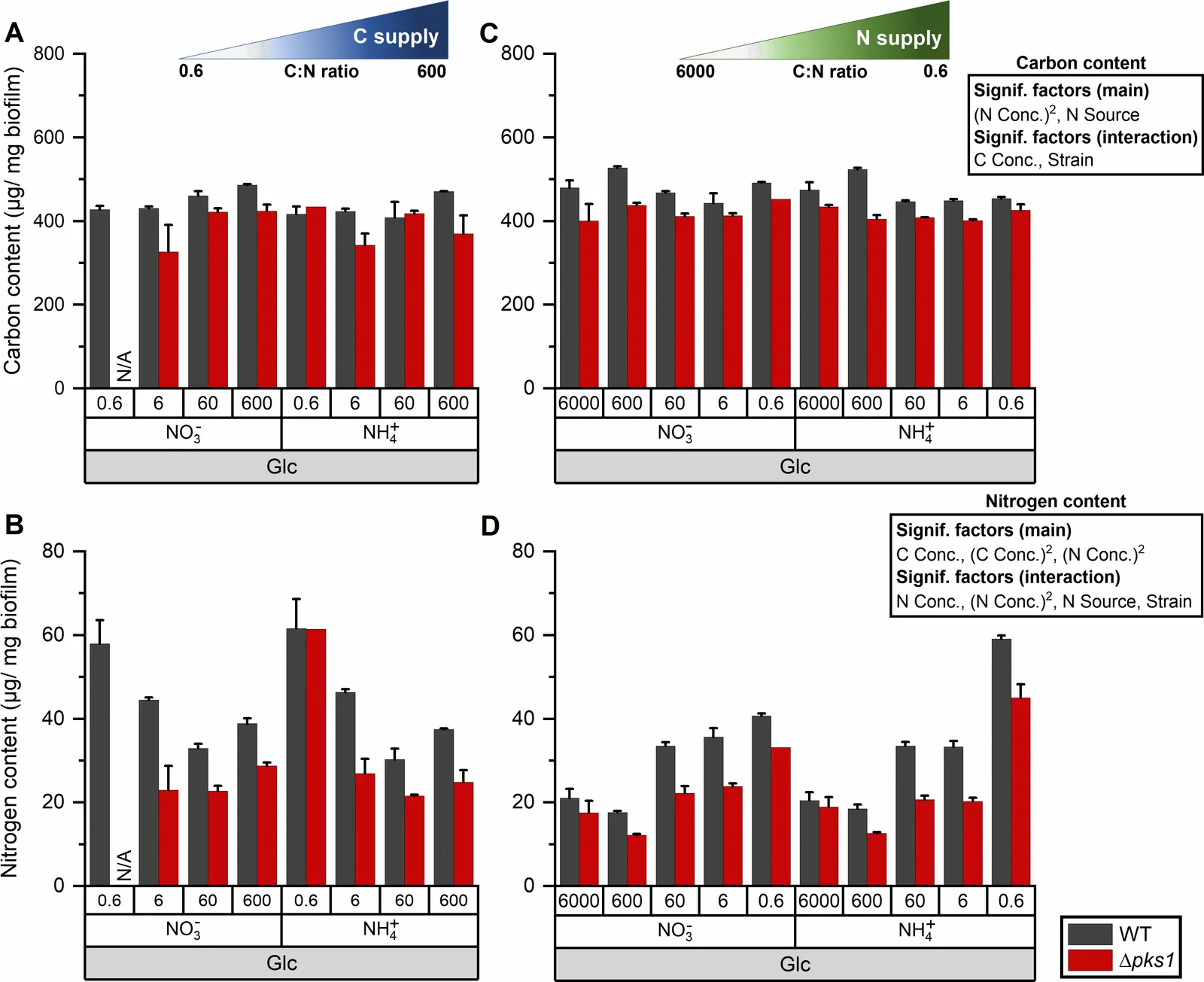
The carbon (**A, C**) and nitrogen (**B, D**) content of the fungal biomass of the WT and ∆*pks1* mutant in the carbon (**A, B**) and nitrogen (**C, D**) experiments. Varying both carbon and nitrogen concentrations in the media had little effect on carbon content but affected the nitrogen content of biofilms; the latter decreased upon increasing the carbon supply or decreasing the nitrogen supply. All experiments were conducted in triplicate, except the carbon experiment with the ∆*pks1* mutant at a C:N ratio of 0.6 with Glc and $NO_{3}^{-}$ due to insufficient growth. Error bars show SE. Statistical significance is shown for the main variables and for the interactions among these variables based on the most parsimonious regression models (Table 2).

The biomass carbon content remained fairly constant upon changing the carbon and nitrogen concentrations, as the differences are significant but relatively small (Fig. 6A and C; Table 2). The WT biomass contained more carbon than the mutant at higher carbon concentrations (Fig. 6C; Table S1).

The biomass nitrogen content, in contrast, was more affected, decreasing at higher carbon or lower nitrogen concentrations (Fig. 6B and D; Table 2). Moreover, upon increasing the nitrogen concentration, the amount of nitrogen in WT biofilms was higher than for the ∆*pks1* (Fig. 6D; Table S1).

The biomass C:N ratio increased with increasing carbon supply to reach an observed optimum around the intermediate C:N ratio of 60. For lower nitrogen supply, the biomass C:N ratio was much higher than in the carbon experiment but dropped to ratios similar to the carbon experiment at higher nitrogen supply. Lastly, the biomass C:N ratio was higher for the melanin-deficient mutant than the WT (Fig. S3; Table 2).

Taken together, the WT biofilms had, for some experiments, higher amounts of carbon and nitrogen compared to non-melanized ∆*pks1* biofilms. The nitrogen content of the biofilm was strongly influenced by changing either carbon or nitrogen concentration, while the carbon content of the biofilm remained relatively stable.

### Carbon use efficiency was less affected by nutrient availability

**Fig 7:**
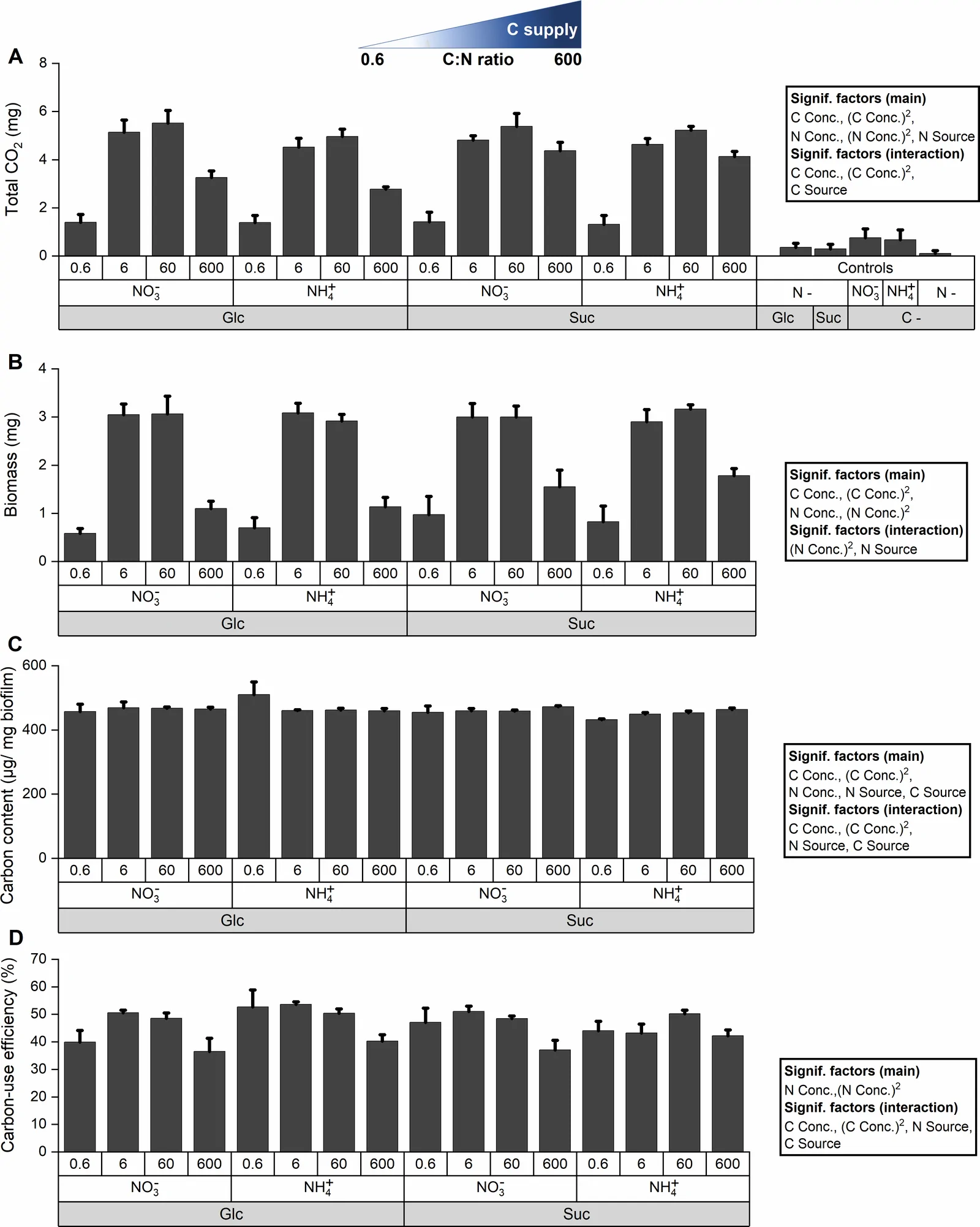
The effect of increasing carbon supply on respiration ($CO_{2}$ production), biomass production, carbon content, and CUE. WT biofilms were grown in 100 mL vials containing 12.5 mL of solid medium at 25°C for 10 days. (**A**) Total $CO_{2}$ (mg) produced over 10 days. Most $CO_{2}$ was produced at intermediate carbon concentrations. Less $CO_{2}$ was produced without nitrogen supplementation than without carbon supplementation. (**B**) Substantially more biomass was produced at intermediate carbon concentrations after 10 days. (**C**) Carbon content of the biomass after 10 days was only slightly increased at higher carbon concentrations. (**D**) CUE varied less than biomass and respiration. All experiments were conducted in triplicate; error bars show SE. Statistical significance is shown for the main variables and for the interactions among these variables based on the most parsimonious regression models (Table 2).

**Fig 8:**
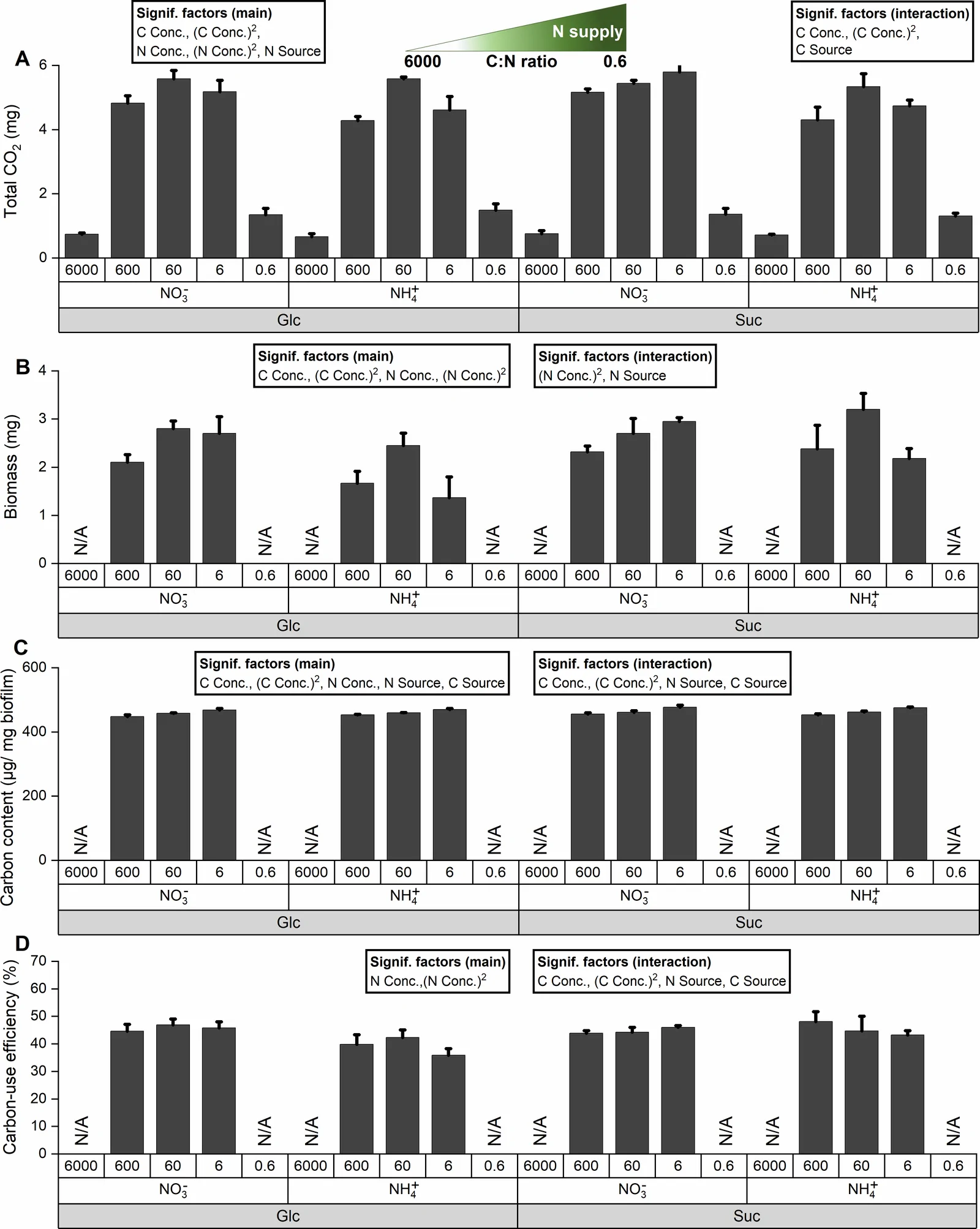
The effect of increasing nitrogen supply on respiration ($CO_{2}$ production), biomass production, carbon content, and CUE. WT biofilms were grown in 100-mL vials containing 12.5 mL of solid medium at 25°C for 10 days. (**A**) Total $CO_{2}$ (mg) produced over 10 days. More $CO_{2}$ was produced at intermediate nitrogen concentrations. (**B**) Biomass production was also highest at intermediate nitrogen concentrations after 10 days. (**C**) The carbon content of the biomass increased slightly with the nitrogen concentration, while (**D**) the CUE showed no clear trend. All experiments were conducted in triplicate; error bars show SE. Statistical significance is shown for the main variables and for the interactions among these variables based on the most parsimonious regression models (Table 2).

The effect of carbon and nitrogen availability on respiration and CUE was determined in closed vials with solid media over 10 days. Respiration, measured as cumulative $CO_{2}$ production, generally peaked around intermediate carbon and nitrogen concentrations (Fig. 7A and Fig. 8A). Biomass formation also peaked around intermediate carbon and nitrogen concentrations (Fig. 7B and Fig. 8B; Table 2). Fungal carbon content showed only minor, though significant, variation across carbon and nitrogen concentrations in the closed-vial system, similar to the open Petri dish experiments (Fig. 7C and Fig. 8C; Table 2). The CUE was also quite similar for all conditions (Table 2). Overall, respiration and biomass formation had clear maxima around intermediate carbon and nitrogen concentrations, while the carbon content and CUE were almost constant.

## DISCUSSION

Although numerous studies have investigated how carbon and nitrogen availability influence the growth of soil fungi, this work is, to our knowledge, the first to systematically dissect the effects of carbon and nitrogen sources and concentrations on an extremotolerant, rock-inhabiting fungus. Since these fungi are considered oligotrophic (18), we hypothesized that they would be able to grow at low carbon and nitrogen supplies and would not have any preferences for specific sources. The use of the model rock-inhabiting fungus *K. petricola* enabled us to also study the effect of melanization by comparing the WT with the melanin-deficient ∆*pks1* mutant. We hypothesized that the production of this carbon-rich polymer would cause a higher sensitivity toward carbon limitation.

*K. petricola* grew to the highest observed extent at the intermediate medium C:N ratio of 60, as indicated by the largest compact growth zone, highest biomass production, thickest biofilm, and highest $CO_{2}$ production. Other studies noted that the optimal observed medium C:N ratio varied considerably among fungal species: Hatakeyama and Ohmasa (52) observed that Boletales species grew best at C:N ratios of 5 to 66, Gao et al. (53) observed that Eurotiales and Hypocreales species grew best at C:N ratios of 10 to 40 and Di Lonardo et al. (37) observed that a species of Mucorales and Hypocreales grew to the highest extent at a C:N ratio of 8. Possible reasons for these observed differences among species include differences in species-specific plasticity of biomass C:N ratios, reduced CUE due to fermentation rather than respiration, use of different carbon and nitrogen sources, or inaccuracies arising from unavoidable limitations in the number of tested C:N ratios. Camenzind et al. (35) found that model-inferred optima for 16 fungal species spanning multiple phyla, including Mucoromycota, Basidiomycota, and Ascomycota, were at medium C:N ratios from 5 to 100, with an average of 47. They moreover noted that this ratio corresponds to twice the C:N ratio of the biomass of known fungal species and proposed that this is due to half the carbon being respired (i.e., a CUE of 50%).

The same reasoning can be applied to our *K. petricola* data. For this fungus, the model-inferred optimal biomass was at the medium carbon concentration of 273 mM and nitrogen concentration of 10.1 mM, i.e., at a C:N ratio of 26.9 (based on all data from the carbon and nitrogen experiments for the WT growing on Glc and averaged for both nitrogen sources, Fig. 5 shows results for $NO_{3}^{-}$). The model-inferred fungal C:N ratio (at this medium C:N ratio of 26.9) was 17.7 ± 2.4 (averaged over both nitrogen sources and the carbon and nitrogen experiments ± the 95% CI). Dividing this by the model-inferred CUE of 0.489 ± 0.031 (at the medium C:N of 26.9 and averaged over the same conditions) gives an estimate of the expected optimal medium C:N ratio of 36.2 ± 5.4 (Table S1). Since growth was not assessed at that medium C:N of 36.2, we need to compare the fungal C:N ratio and CUE-based expected optimum medium C:N ratio of 36.2 with the model-inferred optimal C:N ratio of 26.9. Since this optimum was broad rather than a sharp peak and was inferred from noisy data, it should not be considered to be exactly 26.9 and is thus broadly in line with the expected value of 36.2 ± 5.4. The lower-than-expected model-inferred biomass optimum could, however, indicate that our CUE was underestimated: the expected and model-inferred figures would agree at a CUE of 0.66. Underestimating CUE could be due to loss of carbon through removal of carbon-rich extracellular polymeric substances (EPS) during sample processing or due to CUE increasing over time (we only measured CUE at 10 days). Overall, this suggests that the optimal medium C:N ratio can be largely explained in terms of optimal fungal C:N ratio and CUE.

A variety of other “optima” could be observed in the data (Table S1), but these maxima were more variable and dependent on the nitrogen source and strain. This is likely due to natural selection primarily optimizing biomass growth rather than the partially dependent variables, such as thickness and compact growth zone.

While the compact growth zone, thickness, biomass, and $CO_{2}$ production decreased upon decreasing the supply of either carbon or nitrogen, thus following the Law of the Minimum (54), the filamentous growth zone and penetration depth increased upon lowering the supply of either nutrient. Filamentous growth was most extensive when both carbon and nitrogen were lowest. From the curvature of the fitted relationship (10.5281/zenodo.18980984), filamentous growth would likely increase further at even lower concentrations (which is why “optima” for this response are not given in Table S1) while it was absent around the optimal medium C:N ratio. These results suggest a nutrient scavenging role of filaments, which is also expected to be under direct selection. These observations disprove our hypothesis that this fungus would be less sensitive toward carbon and nitrogen limitations: *K. petricola* behaves like fast-growing fungi *in vitro* (in the absence of physicochemical stresses). Interestingly, the total colony diameter did not change much, indicating that *K. petricola* prioritized extension for foraging over density under nutrient scarcity. Enhanced filamentation under nitrogen limitation is known to be common for fungi (35, 55), but we are not aware of other studies showing enhanced penetration upon nitrogen limitation. Increased filamentation and penetration, however, clearly show that fungi have adapted to scavenge for limiting carbon or nitrogen. The decreased biomass and $CO_{2}$ production under nitrogen limitation fits observations of other fungi (35, 37).

Increasing the carbon and nitrogen supply beyond the C:N ratio of 60 (i.e., a C:N ratio of 600 for the carbon experiment and C:N ratios of 6 and 0.6 for the nitrogen experiment) by adding 1 M of Glc, $NO_{3}^{-}$ or ${NH}_{4}^{+}$ lowered biomass production. This effect was likely caused by osmotic stress, as *K. petricola* growth is reduced upon addition of 1 M NaCl (46).

The nitrogen content increased upon decreasing the supply of carbon or increasing the supply of nitrogen, whereas the carbon and hydrogen contents remained rather stable. The higher flexibility of the nitrogen content compared to the carbon content was also observed for other fungi, both when comparing species or conditions (56–58). At a medium carbon concentration of 273 mM and nitrogen concentration of 10.1 mM (i.e., a C:N ratio of 26.9), the model-inferred biomass C:N ratio of the WT was 17.7 ± 2.4 (95% CI), similar to the mean C:N ratio of 18 derived from a meta-analysis of 377 fungal species (58). These ratios are lower for pathogenic fungi (mean C:N ratio of 13.04), followed by ectomycorrhizal fungi (C:N ratio of 14.44) and saprobic fungi (C:N ratio of 21.19) (58). However, Pánek et al. (57) observed generally lower C:N ratios for saprobes, with soil saprobes having a distinctly lower C:N ratio (i.e., 7.0) than wood saprobes (i.e., 16.3). In general, the value for *K. petricola* therefore aligns best with ectomycorrhizal fungi and wood saprobes, indicating a natural environment characterized by nitrogen limitation.

Our measurements of the carbon and nitrogen content of the biomass did not distinguish intracellular from extracellular carbon and nitrogen (e.g., in EPS or cell wall components), nor whether these represented storage or non-storage compounds. If the increase in nitrogen content under nitrogen surplus was allocated to storage compounds, it would be a type of surplus storage according to Chapin et al. (59). Various carbon storage forms in fungi have been reported, such as triacylglycerides, glycogen, and trehalose (60–62). Nitrogen storage has been studied in filamentous fungi: the rapidly turning over cytosolic nitrogen pool is dominated by glutamine and glutamate, while longer-term, high-density nitrogen reserves consist of basic amino acids (most notably arginine) frequently sequestered in vacuoles (63–66). Moreover, several fungi like *K. petricola* produce EPS, consisting of polysaccharides, proteins, nucleic acids, and lipids, which may play various roles, including facilitating the attachment of colonies to substrata, buffering stresses, and modulating diffusion at the rock-air interface (67–69). EPS have been proposed to function as an external storage pool, a view supported by the ability of *Candida* and *Botrytis* species to use their own EPS as a carbon source (70, 71). However, there is no evidence that RIF can reuse its EPS as a nutrient source.

The carbon source did not significantly influence biomass production, even though growth of other fungi depends on the carbon source being Glc or Suc (72). Depending on the species, fungi metabolize sucrose through extracellular or intracellular hydrolysis (73–75). *Saccharomyces cerevisiae* can hydrolyze Suc both extracellularly and intracellularly (73, 74), whereas some plant pathogenic fungi take up Suc directly from the plant apoplast (75). It is unknown which mechanism is utilized by *K. petricola*, but our observations show that *K. petricola* can hydrolyze sucrose without a noticeable metabolic cost.

The nitrogen source did, however, affect the biofilm morphology: $NO_{3}^{-}$ increased filamentation and penetration at intermediate and higher nitrogen concentrations, respectively. $NO_{3}^{-}$-induced penetration was also observed in *Fusarium oxysporum* (76), *Fusarium graminearum* (77), and *Magnaporthe oryzae* (76). Filamentous and ectomycorrhizal fungi prefer ${NH}_{4}^{+}$ over $NO_{3}^{-}$ (with species-level variability) (78, 79). Because $NO_{3}^{-}$ assimilation requires reduction, which diverts electrons from the respiratory chain and is thus energetically costly, $NO_{3}^{-}$ assimilation can lead to energy limitation. Hence, the enhanced filamentation and penetration under $NO_{3}^{-}$ supply may represent a foraging response where fungi may extend hyphae more aggressively to explore the environment for ${NH}_{4}^{+}$ or other reduced nitrogen sources (79, 80).

Although melanization correlated with more filamentation, it did not affect biomass formation, indicating that melanin did not cause a growth deficit (81). observed the same for *Cryomyces antarcticus*. Since biomass production by the WT was not reduced, we can reject our hypothesis that melanization increases sensitivity toward carbon limitation. The higher nitrogen content of the melanized WT (at higher nitrogen concentrations) is puzzling since the melanin of *K. petricola* is nitrogen-free DHN melanin, but may result from increased EPS production by non-melanized mutants shown previously (69). Another possible explanation is reduced chitin production by the mutant: a meta-analysis of 70 fungal species showed a positive correlation between nitrogen content and chitin content (57). Chitin is essential for retaining/anchoring melanin in the fungal cell wall (82), and inhibition of DHN melanin synthesis in *Alternaria alternata* is accompanied by reduced chitin synthesis (83).

Our observations have some important broader implications. The significant positive relationship between biomass and nitrogen availability suggests that these fungi could act as a pollution indicator, as previously proposed by Krumbein and Gorbushina (84). Furthermore, the increase of the fungal nitrogen content with increasing nitrogen supply suggests that subaerial biofilms mitigate nitrogen pollution in both agricultural (${NH}_{4}^{+}$) and urban ($NO_{3}^{-}$) environments. A similar mitigating effect on nitrogen leaching has been attributed to soil fungi (38).

In conclusion, we show here that carbon and nitrogen limitation affect the growth characteristics of *K. petricola* more than carbon and nitrogen sources and melanization. Surprisingly, our findings for the oligotrophic *K. petricola* generally agree with observations made for fast-growing fungi, suggesting that these characteristics are universal for fungi. Future work could study fungal growth characteristics on the single-cell level or using a larger variety of simple and complex substrates, and including the monitoring of nutrient depletion rates.

## Data Availability

All data supporting the findings of this study are provided within the article and the accompanying supplemental material. The R notebooks and their compiled HTML outputs have been deposited in Zenodo and can be accessed via the DOI 10.5281/zenodo.18980984. The *K. petricola* strains used in this study are available from the authors upon request.

## SUPPLEMENTAL MATERIAL

Supplemental figures aem.00656-26-s0001.pdf Fig. S1 to S3.

Table S1 aem.00656-26-s0002.xlsx Data regression optima.

ASM does not own the copyrights to Supplemental Material that may be linked to, or accessed through, an article. The authors have granted ASM a non-exclusive, world-wide license to publish the Supplemental Material files. Please contact the corresponding author directly for reuse.
